# Room Temperature Tunable Multiferroic Properties in Sol-Gel-Derived Nanocrystalline Sr(Ti_1−*x*_Fe*_x_*)O_3−δ_ Thin Films

**DOI:** 10.3390/nano7090264

**Published:** 2017-09-08

**Authors:** Yi-Guang Wang, Xin-Gui Tang, Qiu-Xiang Liu, Yan-Ping Jiang, Li-Li Jiang

**Affiliations:** 1 School of Physics & Optoelectric Engineering, Guangdong University of Technology, Guangzhou Higher Education Mega Centre, Guangzhou 510006, China; wangyiguang2011@gmail.com (Y.-G.W.); liuqx@gdut.edu.cn (Q.-X.L.); ypjiang@gdut.edu.cn (Y.-P.J.); 2 Laboratory Teaching Center, Guangdong University of Technology, Guangzhou Higher Education Mega Center, Guangzhou 510006, China; jianglili@gdut.edu.cn

**Keywords:** SrTi_1−x_Fe_x_O_3_ thin films, sol-gel, multiferroic, leakage current, conduction mechanism

## Abstract

Sr(Ti_1−*x*_Fe*_x_*)O_3−δ_ (0 ≤ *x* ≤ 0.2) thin films were grown on Si(100) substrates with LaNiO_3_ buffer-layer by a sol-gel process. Influence of Fe substitution concentration on the structural, ferroelectric, and magnetic properties, as well as the leakage current behaviors of the Sr(Ti_1−*x*_Fe*_x_*)O_3−δ_ thin films, were investigated by using the X-ray diffractometer (XRD), atomic force microscopy (AFM), the ferroelectric test system, and the vibrating sample magnetometer (VSM). After substituting a small amount of Ti ion with Fe, highly enhanced ferroelectric properties were obtained successfully in SrTi_0.9_Ti_0.1_O_3−δ_ thin films, with a double remanent polarization (2*P_r_*) of 1.56, 1.95, and 9.14 μC·cm^−2^, respectively, for the samples were annealed in air, oxygen, and nitrogen atmospheres. The leakage current densities of the Fe-doped SrTiO_3_ thin films are about 10^−6^–10^−5^ A·cm^−2^ at an applied electric field of 100 kV·cm^−1^, and the conduction mechanism of the thin film capacitors with various Fe concentrations has been analyzed. The ferromagnetic properties of the Sr(Ti_1−*x*_Fe*_x_*)O_3−δ_ thin films have been investigated, which can be correlated to the mixed valence ions and the effects of the grain boundary. The present results revealed the multiferroic nature of the Sr(Ti_1−*x*_Fe*_x_*)O_3−δ_ thin films. The effect of the annealing environment on the room temperature magnetic and ferroelectric properties of Sr(Ti_0.9_Fe_0.1_)O_3−δ_ thin films were also discussed in detail.

## 1. Introduction

Strontium titanate SrTiO_3_ has been widely applied in electronically tunable microwave devices for its high dielectric, low dielectric losses and high tunability [1,2]. Pure SrTiO_3_ is known as an incipient ferroelectric or paraelectric, since its remaining paraelectric is down to the 0 K under a stress-free condition and it has the instability of ferroelectric at a low temperature [3,4]. A ferrodistortive phase transition temperature from cubic to tetragonal for SrTiO_3_ is as low as 105 K [5,6], which means the ferroelectric properties of the SrTiO_3_ are unavailable in most cases. To obtain the room temperature ferroelectric properties of SrTiO_3_, many efforts have been taken, such as introducing by the strain [7,8,9], substituting the O^16^ with O^18^, or doping with other elements [10,11]. For example, the ferroelectric property has been obtained in the epitaxial SrTiO_3_ film [12] and the large epitaxial strain induced by the lattice mismatch. Besides that, the doping of aliovalent ionic may also be used to provide the strain in the material to promote ferroelectric properties. Among these, the Fe-doped SrTiO_3_ has been proven to be a ferromagnetic [13] with resistive switching characteristics [14], and can also be used as electrode materials [15]. Such characteristics inspire us to investigate the possible multiferroic properties of the Fe-doped SrTiO_3_, which may have potential applications in memory devices, sensors, and actuators [16,17]. Owing to the obtained ferromagnetic property in the Fe-doped SrTiO_3_ [13,18,19,20], and the excellent dielectric properties of SrTiO_3_, the study of the ferroelectric properties will play a significant role on the multiferroic application of the SrTi_1−*x*_Fe*_x_*O_3_.

In fact, besides the strain, the decreasing crystal symmetry and the defects such as oxygen vacancies induced by the Fe ion substitution may also promote the ferroelectric properties in Sr(Ti_1−*x*_Fe*_x_*)O_3_. Thus, the Fe substituting is a quite feasible approach to promote the ferroelectric properties in SrTiO_3_. However, we can find only a little research about the ferroelectric properties of the Sr(Ti_1−*x*_Fe*_x_*)O_3_ films [21,22,23], and the influence of Fe substitution concentration on the ferroelectric and leakage current behavior of the sol-gel derived Sr(Ti_1−*x*_Fe*_x_*)O_3_ thin films has not been reported still now.

In this work, a series of Fe-doped Sr(Ti_1−*x*_Fe*_x_*)O_3−δ_ (STF, *x* = 0, 0.05, 0.1, 0.15 and 0.2; abbreviated as STO, STF05, STF10, STF15, and STF20, respectively) thin films were synthesized on the LaNiO_3_ (LNO) coated Si(100) substrates by the sol-gel method. The multiferroic properties, leakage current behaviors, and conduction mechanism of the STF thin films were investigated. The effect of annealing environment on the room temperature magnetic and ferroelectric properties of Sr(Ti_0.9_Fe_0.1_)O_3_ thin films were also discussed in detail.

## 2. Results and Discussion

**Figure 1:**
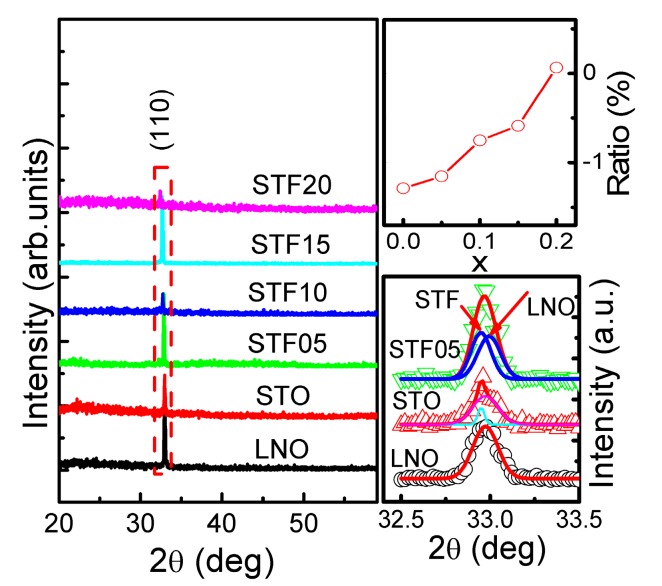
X-ray diffraction (XRD) patterns of the Sr(Ti_1−x_Fe_x_)O_3_(STF) thin films grown on the LaNiO_3_ (LNO) buffered Si(100) substrates. The top inset shows the variation ratio of the in-plane lattice parameters *a* with respect to the bulk STO material; (*a* − *a*_0_)/*a*_0_, the bottom inset, shows the magnified plot of the (110) peaks for the LNO and STF.

The X-ray Diffraction (XRD) patterns of the Sr(Ti_1−*x*_Fe*_x_*)O_3_ (STF) thin films grown on the LaNiO_3_ (LNO) buffered Si(100) substrates were shown in Figure 1. The (110) peak responsible for the perovskite structure was observed in all film samples, showing a typical polycrystalline perovskite nature of the STF films with a highly preferential (110) orientation. The orientation can be ascribed to the quite similar lattice constant and crystal structure of the LNO and STF thin films, where the LNO can be used as a seeding layer for favoring the nucleation and growth of the STF films [24]. The XRD peaks shift to a lower diffraction angle with increasing Fe concentration, which is the result of increased lattice parameters. The lattice parameters are 3.855, 3.860, 3.876, 3.882, and 3.908 Å, respectively, for Sr(Ti_1−*x*_Fe*_x_*)O_3_ thin films with *x* = 0 to 20. This increase of lattice parameters with *x* is attributed to the increasing of low-valence-state Fe ions [25]. The various ratios of the lattice parameters *a* with respect to the bulk STO material (cubic: *a*_0_ = 3.905 Å), i.e., (*a* − *a*_0_)/*a*_0_, are presented in the inset of Figure 1. The compressed lattice parameter of thin film compared to the bulk material (ratio < 0) indicates the existence of strain in the film. The magnified plot of the peaks was also presented in the inset of Figure 1, which indicates the superposed peak of the LNO (110) and STF (110) clearly. 

**Figure 2:**
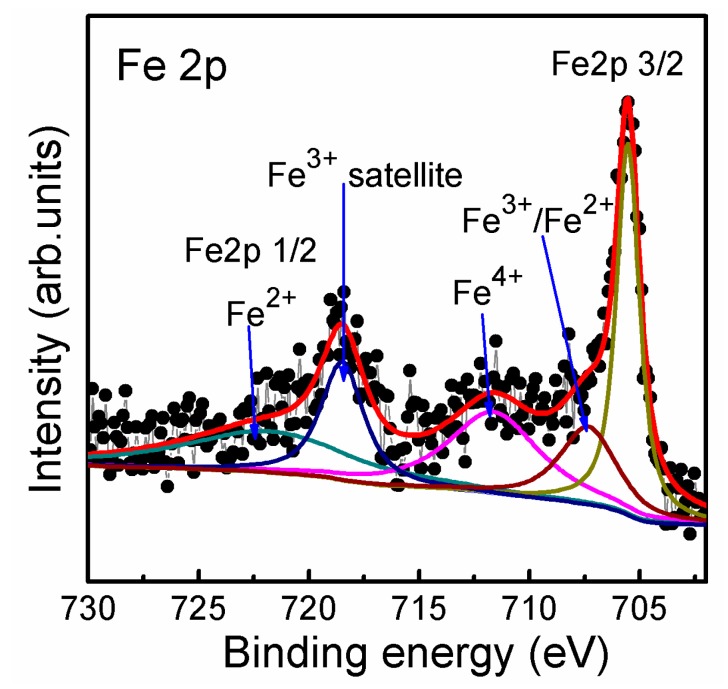
Fe 2p X-ray photoelectron spectroscopy (XPS) splitting spectrum of the STF10 thin film on LNO/Si(100) substrate.

The valence states of the Fe ions in STF10 thin film characterized by X-ray photoelectron spectroscopy (XPS) (Thermo Fisher Scientific Inc., Waltham, MA, USA) were shown in Figure 2. The composition of STF10 thin film was analyzed by XPS as well. The atomic ratio of Sr:Ti:Fe was found to be 10.35:10.49:2.35, which slightly deviated from the theoretical value of 10:9:1 of the stoichiometric thin film. Besides the analytical error, the deviation may be relevant with the presence of divalent Fe ions. Since the ions can be incorporated in the SrO sublattice, higher Fe concentration may indicate its migration towards extended defects and the surface [26], which is, however, not sufficient to kill the long range atomic order. The Fe 2p 2/3 and 2p 1/2 doublets of STF10 were seen in the vicinity of 706 eV (705.5, 707.3 eV) and 721.76 eV, respectively. These peaks appear at a lower binding energy compared to measurements on Fe_2_O_3_ with Fe^3+^ [13], implying the existence of Fe^2+^ and Fe^3+^ in STF10. The clear Fe^3+^ satellite peak was present at 718.48 eV. The peak at 711.6 eV is about 0.9 eV higher than the Fe 2p 2/3 of Fe_2_O_3_, indicating the possible existence of Fe^4+^. Thus, the XPS result shows the coexistence of Fe^2+^, Fe^3+,^ and Fe^4+^ mixed valence states in STF10 thin film, with dominance of the Fe^3+^ states, which is consistent with the previous works and corresponds to the existence of oxygen vacancies [13].

**Figure 3:**
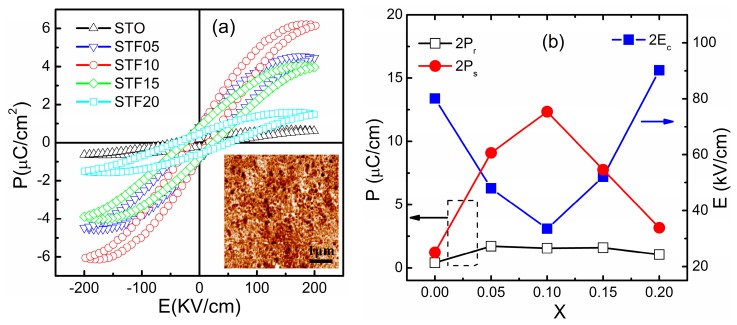
Ferroelectric properties and morphology of the STF thin films: (**a**) electric polarization (*P*) as a function of the electric field (*E*) for the STF thin films. The inset is the atomic force microscopy (AFM) image of the STF10 thin film; (**b**) the variation values of the remnant polarization (*P_r_*), saturation polarization (*P_s_*), and the coercive field (*E_c_*) as a function of the Fe concentration *x*.

The morphology of the STF10 thin film was displayed in the inset of Figure 3a, which exhibits a dense micro-structure with no cracks. The calculated results of the atomic force microscopy (AFM) (Being Nano-Instruments Ltd. Beijing, China) image showed that the average grain size is about 86 nm and the root mean square roughness of the STF10 thin film is 5.8 nm. Such nanoscale grains were the result of the rapid thermal annealing (RTA) process, which can restrain the grain growth effectively.

The electric-filed-induced polarization (*P*-*E*) switching behavior measurements under 1 kHz at room temperature were shown in Figure 3a. It is observed that all samples showed an almost perfect symmetrical *P*-*E* loop along both the electric field axes and the polarization axes, indicating the existence of ferroelectric properties. The rather low values of saturation polarization (*P_s_*) and remnant polarization (*P_r_*) of STO imply a lack of obvious ferroelectric signal in the STO thin film, which is consistent with the nature of incipient ferroelectric, and the weak signal can be interrupted as the result of internal strain. When substituting the Ti ion with the Fe ion, the ferroelectric properties of the thin film improved significantly. The variation values of *P_r_*, *P_s_*, and the coercive field (*E_c_*) with the concentration of Fe are shown in Figure 3b. With increasing Fe concentration *x* (when *x* ≤ 0.1), the value of 2*P_s_* increases and reaches a maximum of 12.34 μC·cm^−2^ when *x* = 0.1. However, upon a further increase in Fe concentration (when *x* > 0.1), the 2*P_s_* decreases instead. Slightly different, the maximum of 2*P_r_* was obtained with 2*P_r_* of 1.71 μC·cm^−2^ for *x* = 0.05. 

**Figure 4:**
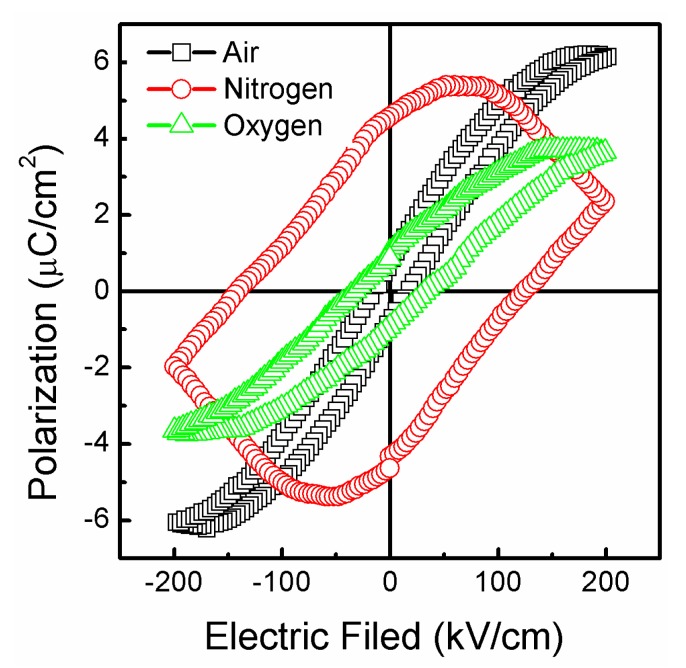
Ferroelectric properties of the STF10 thin films were annealed at various atmospheres.

Figure 4 shows the *P*-*E* loops of the STF10 thin films annealed at various atmospheres. When the thin film samples are annealed at air and oxygen atmospheres, the values of the 2*P_r_* and 2*E_c_* are 1.56 μC·cm^−2^ and 32.0 kV·cm^−1^, and 1.95 μC·cm^−2^ and 65.1 kV·cm^−1^, respectively. The ferroelectric properties of the thin films were annealing at oxygen atmosphere (which is better than that of the samples that were annealed at air atmosphere). When the sample was annealing in a nitrogen atmosphere, the values of the 2*P_r_* and 2*E_c_* were 9.14 μC·cm^−2^ and 265.4 kV·cm^−1^, respectively. The 2*P_r_* value of the nitrogen atmosphere annealing sample was the best; the major source was from the leakage current. The 2*P_r_* value (9.14 μC·cm^−2^) of the STF films is higher than that of tetragonal strontium titanate thin films on SrTiO_3_ (001) substrates (2.5 μC·cm^−2^) [27], higher than that of tensile-strained SrTiO_3_ thin films on the GdScO_3_ (110) substrate (0.5 μC·cm^−2^) [28], and compares with the etragonally strained SrTiO_3_ thin films on the single crystal Rh substrate (8 C/cm^2^) [29], respectively, at room temperature.

Although the cause of improved ferroelectric properties in STF thin films is complicated, there are primarily three possible factors that influence the ferroelectric properties in this system. One is the internal strain induced by the misfit of the substrate and ionic substitution of Fe, the other is the decrease of crystal symmetry induced by the aliovalent Fe ion and oxygen distributions; both of them are beneficial for the enhancement of spontaneity [30]. The last is defect-induced by the Fe^3+^ or Fe^2+^ ion substitution. For instance, in the typical case of STF10, the existence of Fe^3+^ has been confirmed by the XPS results, and the possible Fe^2+^ also been observed. The Ti ion usually presents a valence of +4 in STO, when substituting that ion with the Fe ion in the STO lattice, oxygen vacancies (V_Ö_) are generally created in order to maintain the charge balance [31]. When the concentration of Fe is above a degree, the defect dipoles of the cations (i.e. Fe ions)-V_Ö_ complex could exist in the unit cells by binding cations and oxygen vacancies [32,33], and therefore increase the total polarization [34]. However, meanwhile, the doping of Fe ions can also weaken the ferroelectric of the STF thin films because of the strong magnetic coupling between the doped Fe ions [18]. Therefore, the polarization did not increase linearly with the increasing concentration of Fe. The 2*E_c_* showed a completely opposite trend with the variation of 2*P_s_*, which is consistent with the general variation trend of ferroelectric properties. The change of *E_c_* could arise from the change in domain switching dynamics, and can similarly be explained as the result of changed crystal symmetry and defect dipoles induced by the Fe-doping. 

**Figure 5:**
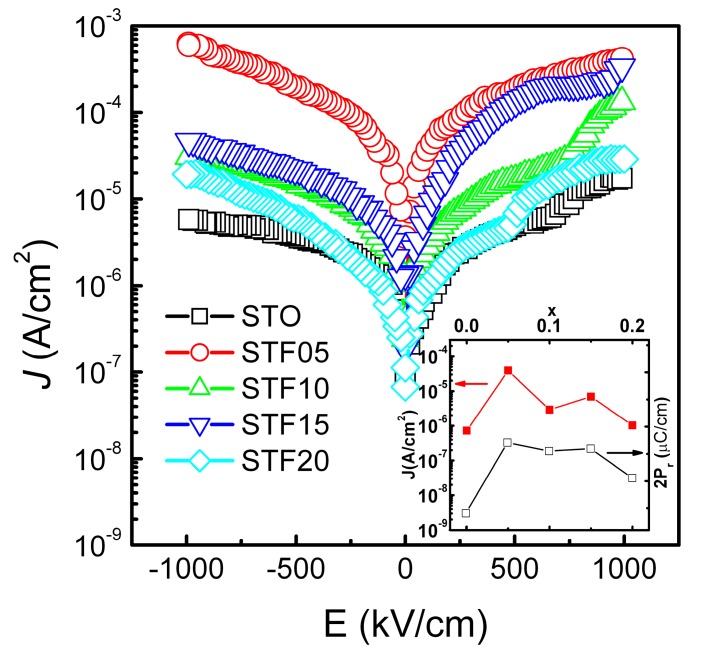
Leakage current densities (*J*) as a function of electric field (*E*) for STF thin films. Note the negative voltage axis was used. The inset shows *J* at applied field of 100 kV/cm and the values of double remnant polarization (2*P_r_*) varying with the concentration of Fe.

The leakage current densities (*J*) as a function of the electric field (*E*) for the STF thin films were shown in Figure 5. The Inset illustrates the influence of the Fe doping on the leakage current densities of the STF thin films. After substituting the Ti with Fe, the leakage current densities of the STO thin film increased from 7.22 × 10^−7^ A·cm^−2^ to about 10^−6^–10^−5^ A·cm^−2^ at an applied field of 100 kV·cm^−1^. The effect could be attributed to the oxygen defect induced by the mixed-valence Fe substitution, where Fe ions can shape the energy-band structure of STF, resulting in a decrease in band-gap energy, reduction enthalpy, and increasing levels of disorder in the oxygen sublattice [35], therefore increasing the free carriers in the films. On the other hand, since oxygen vacancies can be used as trapping centers for electrons, shallow trap energy levels may be generated within the band gap for the mobility of activated electrons. This is similar with the case in Ni-doped BiFeO_3_, where the increased leakage was caused by the oxygen vacancies [36]. However, due to the possible changes in the oxidation states of the Fe ions and the decrease of crystal asymmetry, the oxygen vacancies will not always increase with the increasing Fe concentrations [37]. In addition to the bulk limited conduction, the significant difference in leakage when the bias reversed indicates the effect of interface limited conduction [38]. Thus the leakage current density did not exhibit a linear increasing trend with the increasing Fe concentration. The consistent variation of the *P_r_* and *J* indicates the possible contribution of *J* to the ferroelectric properties of STF thin films, most probably through the oxygen vacancies and the defect dipoles. Though the difference between the pure STO and STF thin films is about a 1–2 order of magnitude, the *J* in the films is low enough compared to that of the atomic layer deposition pure STO films [39], which indicates a potential application in dielectric devices.

**Figure 6:**
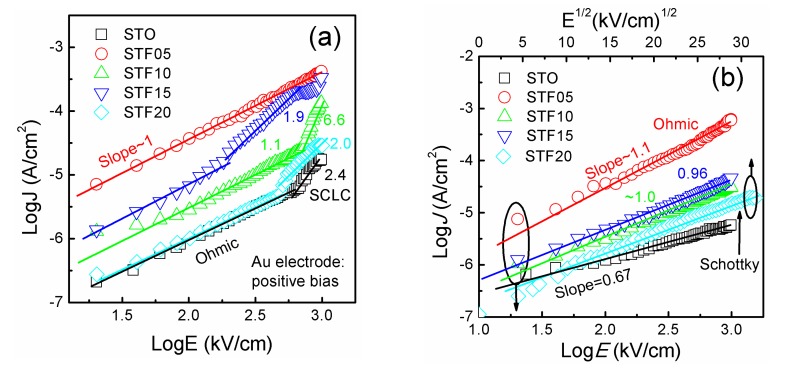
log*J* versus log*E* (or *E*^1/2^) plot of the STF thin films when (**a**) positive bias (**b**) negative bias is applied on the Au electrode, respectively. The red lines show the fitted linear segments of the plots.

Several models were applied in order to investigate the conduction mechanism of the films in detail, and the typical fitted curves were shown in Figure 6. Figure 6a,b shows the log*J* versus log*E* (or *E*^1/2^) plot of the STF thin film with the positive and negative bias on the Au electrode, respectively. In the case of the STO thin films with the positive bias on the Au electrode, see Figure 6a, the log*J* versus log*E* plot in low field region (*E* < 700 kV·cm^−1^) was fitted well by the linear segment with slope ~1, which indicates the Ohmic conduction behavior [40,41]. The same behavior was also observed for many other samples, especially in the low field regions. In the high field region (*E* > 700 kV·cm^−1^) of the STO thin film, as seen in Figure 6a, the slope of the log*J* versus log*E* plots is larger than 2, which reveals a space-charge-limited conduction (SCLC) [42]:

(1) $$J=\frac{9\varepsilon_{0}\varepsilon_{r}\mu \theta E^{2}}{8d}$$

where *ε*_0_ is the permittivity of free space, *ε_r_* is the low-frequency permittivity of the film, *μ* is the charge carrier mobility, *θ* is the ratio of the free carriers to the total carriers, and *d* is the thickness of the film. The conduction mechanism also predominated in the high field region (*E* > 490 kV·cm^−1^) of the STF10, STF15, and STF20 thin films. Different from that, for the negative bias region of the STF20 thin films, the log*J* showed a linear relationship with the *E*^1/2^ (Figure 6c,d), indicating the Schottky emission conduction mechanism [43]:

(2) $$J=A^{*}T^{2}\exp\left[-\frac{1}{k_{B}T}\left(\Phi_{B}-{\left(\frac{q^{3}E}{4\pi \varepsilon \varepsilon_{0}}\right)}^{\frac{1}{2}}\right)\right]$$

where A* is the Richardson constant, and Φ_B_ is the Schottky barrier height. Here, barriers are induced by the contact of Au and STF20. 

The conduction mechanisms for all samples in the full region were listed in the Table 1. In the low field regions, most thin films showed Ohmic conduction behavior, and the SCLC mechanism was observed in higher field regions. This is because the SCLC will not be observed until the injected free-carrier density exceeds the volume-generated, free-carrier density [44]. In this way, the transition field can indicate the level of volume-generated, free-carrier density indirectly. We thus can speculate that the STF10 and STF15 thin films possess a higher volume free-carrier than the STO thin film, because the transition field from Ohmic to the SCLC mechanism for STO is lower, and the three thin films have the same conduction mechanism in other regions. This is exactly consistent with the experiment result, where the *J* in STF10 is higher than that of STO and STF15 possess a higher *J* than that of STF10. As discussed above, after Fe substitution, oxygen vacancies were generated and the energy-band structure of STF was shaped with a reduced band-gap energy and reduction enthalpy, resulting in the increase of the free carriers in the films. A further increase of *x* (*x* = 0.20) leads to the significant effect of interface-limited Schottky emission. The mechanism arises from the difference in Fermi levels between the electrode and the thin films, which will create a potential barrier to limit the *J*. It is likely that the doped Fe ion has influenced the barrier through a changing concentration of defects and therefore affects the Fermi level, which is consistent with the pervious study that Fe substitution can change the energy-band structure of STF [31]. Also, based on the study, the defect concentrations of bulk STF were determined by the balance between the intrinsic electronic and ionic disorder and the redox reaction. Moreover, owing to the existence of the interface in the thin films, the defects and carrier concentrations are more difficult to study, and more works are needed to test the specific details.

**Figure 7:**
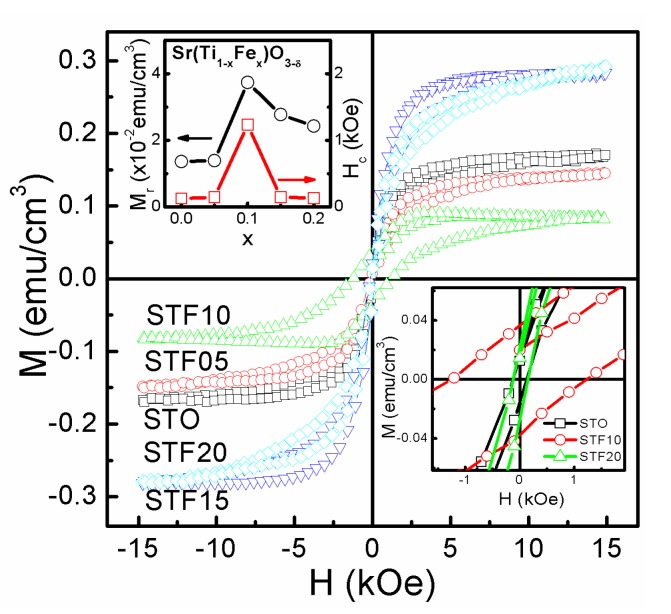
Magnetization-magnetic field (*M*-*H*) curves of STF thin films measured at room temperature. The inset shows the magnified plot of the vicinity “0” magnetic field (**bottom**) and the variation values of the remanent magnetization (*M_r_*) and coercive magnetic field (*H_c_*) as a function of the Fe concentration *x* (**top**).

Figure 7 shows the magnetization-magnetic field (*M*-*H*) curves of the various thin films on LNO/Si(100) substrates at room temperature by applying an in-plane magnetic field. The top inset shows the magnified plot of the vicinity “0” magnetic field. In the cases of STO, STF10, and STF20, the thin films exhibits weak ferromagnetism with average remnant magnetization (*M_r_*), saturated magnetization (*M_s_*), and a coercive magnetic field (*H_c_*) of 1.46 × 10^−2^ emu·cm^−3^, 1.62 × 1^−1^ emu·cm^−3^, and 123 Oe for STO, 3.74 × 10^−2^ emu·cm^−3^, 9.22 × 10^−2^ emu·cm^−3^, and 1231 Oe for STF10, and 2.43 × 10^−2^ emu·cm^−3^, 2.33 × 10^−1^ emu·cm^−3^, and 136 Oe for STF20, respectively. The weak room temperature ferromagnetism in SrTiO_3_ thin films was usually attributed to the surface defects, such as oxygen vacancies, cation vacancies, oxygen-ended polar terminations, and the effects of the grain boundary [45,46,47,48,49,50,51]. For the STF thin films, magnetoelastic effects may be an important contributor to the obviously enhanced ferromagnetic properties. In transition-metal-substituted oxides thin films, lattice mismatch, thermal mismatch, and coalescence during growth typically lead to strain. Also, magnetoelastic effects can be associated with the magnetic behaviours in the strain thin films, which have been investigated in some transition-metal-substituted STO films [52]. Based on the preceding discussions, the surface defects such as oxygen vacancies, strontium vacancies, iron vacancies, titanium vacancies, and oxygen-ended polar terminations [51] are perhaps the most probable magnetic sources, all of them being mainly located at the surface of the nanograins. A series of papers have proposed the grain boundaries as the controlling factor for the ferromagnetic behavior of oxides [47,48,49,50,51]. The bottom inset of Figure 7 (lower right) shows the variation of average *M_r_* and *H_c_* as a function of *x*. The *M_r_* and *H_c_* values of the thin films decrease when the Fe concentration *x* is higher than 10%. The phenomenon may be caused by the antiferromagnetic coupling between the Fe ions, which can only be exhibited as the Fe concentration increases to a certain degree [18].

**Figure 8:**
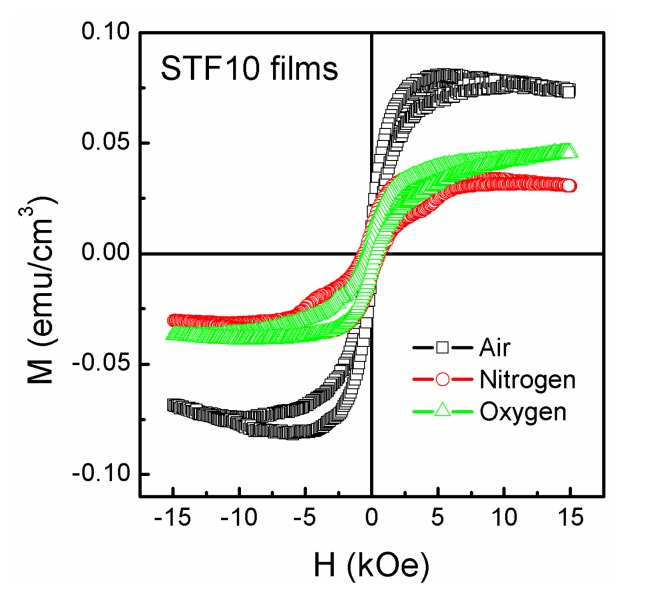
Room temperature *M*-*H* curves of the STF10 thin films were annealed at various atmospheres.

Figure 8 Room temperature *M*-*H* curves of the STF10 thin films were annealed at various atmospheres. In the case of the various atmospheres annealing, the thin films exhibit weak ferromagnetism with average values of the *M_r_*, *M_s_*, and *H_c_*, which are 1.21 × 10^−2^ emu·cm^−3^, 8.83 × 10^−2^ emu·cm^−3^, and 203 Oe for air atmosphere, 1.09 × 10^−2^ emu·cm^−3^, 4.01 × 10^−2^ emu·cm^−3^, and 613 Oe for nitrogen atmospheres, and 7.40 × 10^−3^ emu·cm^−3^, 3.77 × 10^−2^ emu·cm^−3^, and 359 Oe for oxygen atmosphere, respectively. Compared with the ferroelectric properties, the sample shows the largest remanent magnetization when it is annealing in air atmosphere and showing good ferromagnetism. The samples show relatively weak ferromagnetism when it was annealed in oxygen or nitrogen atmosphere. Among them, the sample of nitrogen annealing also has less magnetic susceptibility in the case of the high magnetic field, showing a certain diamagnetism. Therefore, the air annealed sample with the higher oxygen vacancy concentration and grain boundaries exhibits good ferromagnetism. However, when the concentration of the oxygen vacancy is too high, the antiferromagnetic phase appears in the sample, which shows certain diamagnetism in the sample. Similar to ferroelectricity, the ferromagnetism shows a significant change in vacancy concentration, which further verifies that the ferromagnetic properties of the samples are closely related to the mixed valence ions and the effects of the grain boundary in the samples [47,48,49,50,51].

## 3. Materials and Methods

Sr(Ti_1−*x*_Fe*_x_*)O_3_ (STF, *x* = 0, 0.05, 0.1, 0.15, and 0.2; abbreviated as STO, STF05, STF10, STF15, and STF20, respectively) thin films were synthesized on the LaNiO_3_ (LNO) coated Si(100) substrates by a sol-gel route with a spin-coating process [23]. To form the precursor, reagent-grade strontium acetate, iron nitrate nonahydrate, and titanium butyrate were dissolved under continuous stirring at 60 °C in solvents acetic acid, 2-methoxyethanol, and acetyl acetone, respectively. Here, acetyl acetone was also used to stabilize titanium butyrate. The three solutions were mixed and then stirred together at 60 °C for 1.5 h, forming a complete homogeneous transparent solution. The concentration of the final solution was adjusted to 0.25 M with a pH value of 2–3 by adding 2-methoxyethanol and acetic acid. Prior to spin-coating, the solution was filtered to avoid particulate contamination. The LNO thin layer prepared by chemical precursor solutions was described in previous literatures [24]. The STF layers were spin-coated onto the LNO films at a speed of 4000 rpm for 60 s. After each spin coating process, samples were heat-treated at 300 °C for 1 h on the hot plate. The step is repeated twice to obtain the desired thickness of the STF thin films. The STF films on LNO coated Si(100) substrates were finally annealed at 650 °C for 15 min by rapid thermal annealing (RTA) in air. To compare the multiferroic properties of the STF thin films annealing at various atmospheres, the STF10 thin films samples were selected annealing at 650 °C for 15 min by RTA in air, oxygen, and nitrogen atmospheres, respectively. As measured by a surface profiler (KLA-Tencor P-10, ClassOne Equipment, Inc. Decatur, GA, USA), the mean thickness of the annealed LNO and STF films was about 80 nm and 100 nm, respectively. 

The crystalline phase and micrograph of the STF thin films were identified by the X-ray diffractometer (XRD, Pgeneral XD-2, PERSEE, Beijing, China) using CuKα radiation and atomic force microscopy (AFM, BenYuan CSPM-5500, Being Nano-Instruments Ltd. Beijing, China), respectively. The surface chemical states of the thin film were characterized by X-ray photoelectron spectroscopy (XPS, Thermo Scientific ESCALAB 250, Thermo Fisher Scientific Inc., Waltham, MA, USA) with the Al Kα radiation source. To investigate the electrical properties of the STF thin films, the gold (Au) top electrode with a diameter of 0.2 mm were deposited on the surface of the STF films by a vacuum evaporation apparatus (KYKY SB-12, KYKY Technology Co., Ltd. Beijing, China) through a shadow metal mask. The ferroelectric properties and the leakage current characteristics were measured by a ferroelectric test system (Radiant Precision Premier II, Albuquerque, NM, USA). Magnetic properties of the films were measured by a vibrating sample magnetometer (VSM, PPMS-9, Quantum Design, San Diego, CA, USA) at room temperature.

## 4. Conclusions

SrTi_1−*x*_Fe*_x_*O_3_ (0 ≤ *x* ≤ 0.2) thin films have been synthesized on the LNO-coated Si(100) substrates by the sol-gel technique. The ferroelectric properties of the STO thin film has been improved through Fe doping, with a maximum double saturated polarization (2*P_s_*) of 12.34 μC·cm^−2^ when doped with 10% Fe. The strain, crystal asymmetry, and defect dipoles induced by the ion substitution were ascribed to the possible origin of the enhanced ferroelectric properties. The leakage current densities of the Fe-doped STO thin films are about 10^−5^–10^−6^ A·cm^−2^ at an applied field of 100 kV/cm, about 1–2 orders of magnitude larger than that of the pure STO thin film. The conduction mechanism of the thin films with various Fe concentrations has been discussed in detail. The oxygen vacancies are concluded to play a significant role on the conduction properties of the thin films. The ferromagnetic properties of the STF thin films have been investigated. The mixed valence ions and effects of the grain boundary were used to explain the room temperature ferromagnetism. The obtained low current densities in the films allow the possible application in multiferroic and electronic devices.

## Figures and Tables

**Table 1:** The conduction mechanism of the STF thin films annealed at 650 °C in air.

Samples	STF/LNO Interface				Au/STF Interface
	Low Field (kV/cm)	Mechanism	High Field (kV/cm)	Mechanism	Mechanism
STO	<700	Ohmic	>700	SCLC	Ohmic
STF05	-	Ohmic	-	Ohmic	Ohmic
STF10	<710	Ohmic	>710	SCLC	Ohmic
STF15	<120	Ohmic	>120	SCLC	Ohmic
STF20	<490	Ohmic	>490	SCLC	Schottky
